# ‘e’-thinking teaching and assessment to uphold academic integrity: lessons learned from emergency distance learning

**DOI:** 10.1007/s40979-021-00079-5

**Published:** 2021-08-24

**Authors:** Zeenath Reza Khan, Shivadas Sivasubramaniam, Pranit Anand, Ajrina Hysaj

**Affiliations:** 1 Faculty of Engineering and Information Sciences University of Wollongong in Dubai, PO Box 20183, Dubai, UAE; 2 Human Sciences Research Centre, School of Human Sciences, University of Derby, Kedleston Road, Derby, DE22 1GB UK; 3 Learning and Teaching Unit, Queensland University of Technology, Gardens Point, Brisbane, Queensland Australia; 4 UOWD College, University of Wollongong in Dubai, PO Box 20183, Dubai, UAE

**Keywords:** Assessment design, Redesign, Technology, Smart education, Academic integrity, Pandemic

## Abstract

Covid-19 pandemic had an impact on many day-to-day activities but one of the biggest collateral impacts was felt by the education sector. The nature and the complexity of higher education is such that no matter how prepared we are as faculty, how planned our teaching and assessments, faculty are all too aware of the adjustments that have to be made to course plans, assessments designed, content delivery strategies and so on once classes begin. Faculties find themselves changing, modifying and deviating from original plans to ensure accessibility and inclusiveness, this may be due to a variety of reasons such as student abilities, behaviour, disturbances and even outside factors that may be political, environmental, social etc. Majority of the time, faculty are prepared for the change that needs to be incorporated and are quick to adjust. However, no one expected the disruption to education that was caused by COVID19 pandemic. The world came to a standstill while schools and universities scrambled to push learning to the digital space. It was important to try to ensure continuity of learning for students, but the issue of integrity came to the forefront by summertime. Faculties were suddenly expected to restructure their lessons, delivery, teaching and assessing digitally, at the same time ensuring and upholding integrity of the concepts taught and assessed. This has neither been easy or straightforward because the situation was unprecedented with little or no prior documentation or guidelines to help. Recognising this gap, this paper is an attempt at providing exploratory findings from authors’ experiences in their respective institutions over the ensuing months. The paper attempts to record the changes made by the faculty and colleagues to lessons and assessments with particular focus on how technology has been used to help restructure classes, deliver lessons and assess students which have aided in minimizing the likelihood of students cheating. The paper further narrates the reflective changes that were made in response to experience, student/external examiners feedback etc.

## Introduction

Continuing education for students was one of the top most priorities for a large portion of countries when COVID19 peaked in March–April 2020. With over 1.6 billion students out of classrooms due to lock down measures by governments, many countries scrambled to move the learning and assessments online to help continue education for the students (UNESCO, 2020). This became known as emergency distance learning, a treatment rather than a strategy in response to the pandemic taking over.

Many educational institutions struggled with the move to online platforms, such as lack of training, lack of resources, lack of funds, lack of buy-in from staff and students to name a few. By the end of the term, it was obvious that COVID19 was not going anywhere and institutions now had to think about assessments, particularly end of year/term assessments. Amidst the chaos of lockdowns, saving lives and keeping minimum necessity functions going, governments also faced backlash from student bodies when online proctoring software became prevalent across schools and universities. Finding a balance between positive learning and upholding academic integrity became a tug of war.

This paper uses experiential research method (Grant et al., 2001) to reflect authors’ experiences and shared conversations on the topic of integrity during the pandemic; attempts to identify some barriers to maintaining integrity and through experiences explores the various technological interventions to teaching, learning and assessing introduced or continued in their classes and how they may have helped minimize students’ likelihood to commit misconduct during the pandemic’s emergency distance learning.

This paper has been arranged as follows: the paper looks at assessment design and its role on teaching and learning; it then provides a brief overview of technology in education, and its role in upholding integrity. The paper then proposes a methodology followed by results, shared analysis and discussion, and conclusion.

### Assessment design and its role in teaching and learning and upholding integrity

Studies have shown that assessment design cannot completely eradicate the problem of academic misconduct, such as plagiarism however, there is enough evidence to suggest that some types of assessments are harder for students to plagiarise, and then certain assessment designs may actively discourage plagiarism and other acts of misconduct through engagement and a focus on learning rather that the final grade (Ellis, et al., 2020; Jansen & Nicole, 2017). These types of assessments emphasize “learning for skills application” rather than “learning for knowledge reproduction”. Pedagogical approaches have recognized active learning as a focal point for achieving high levels of student’ satisfaction and respectively increased levels of faculty professional satisfaction. Students’ academic satisfaction with the process of learning is interconnected with the faculty satisfaction with the process of teaching. As educators, we design curriculums aiming at the achievement of common goals of teaching and learning. However, we are all quite aware of the necessity to consider students’ perspectives on learning alongside the demands imposed by the learning outcomes and relationship between general subjects or subjects of different disciplines (Phillips, 2005).

Students’ active learning has been considered a crucial element in encouraging students to be more honest and has been analysed for the last two decades (Walker, 2003; Settles and Craven, 2008; Roehl et al., 2013 and McConnell et al., 2017). Active learning is seen as a well-suited solution to many challenges related to development of critical thinking, participation in group work and most importantly to the designing of authentic assessments that aim for creation and submission of reliable and a high standard work from students individually, in pairs or in groups. According to Settles and Craven (2008) and Hysaj et al. (2018) higher education utilizes multiple corpora that aim creation and utilization of novel procedures to demonstrate and analyze strategies of development of active learning; while addressing the importance of critical thinking and recognizing the limitations of the learning outcomes, curriculum design or the assessment tasks.

Development of critical thinking is not only crucial in higher education but it is essential in all aspects of life. Assessment tasks generally evaluate critical thinking when addressing the variety of solutions provided by students, as well their logicality and the coherence of writing (Şendağ and Odabaşı, 2009; Sharov et al., 2019; Hysaj and Hamam, 2020). Educators in higher education facilitate the process of developing critical thinking through problem-solution assessment tasks, group work assessment tasks (Gleason et al., 2011; Lee et al., 2020) that improve students’ cultural knowledge, as well as through lectures and tutorials that challenge students understanding of given topics through addressing possible bias (Lombard and Grosser, 2008). Although educators commonly aim at the presence and the disposition of students to think critically, the latter may consider this as an added challenge, hence aiming instead to merely complete assessment tasks without any meticulous consideration of all the aspects of design and completion (Walker, 2003). Therefore, enabling students’ development and demonstration of students’ critical thinking requires inclusiveness of the same in the lectures, tutorials, case studies, debates and assessment tasks.

Students may at times feel intimidated by the lack of critical thinking or the application of it when working in groups or attending lectures. Therefore, it could prove to be very encouraging to involve them in the process of searching for information through assigned tasks that require investigation of solutions and approaches (Park and Lee, 2017), to help them become confident in their own work and abilities, rather than look for support elsewhere .

### Technology in teaching, learning and assessing and its role in upholding integrity

Sir Isaac Pitman mailed postcards with texts transcribed into shorthand as part of his attempt to teach shorthand to his students in 1840 (Bates, 2016). Distance learning or correspondence courses have always been popular and gained momentum with the advent of the Internet and online learning.

Using technology in classrooms for teaching and assessing has been making its presence felt for decades, not just helping teachers to blend their delivery but also ways to assess them and going beyond to even provide complete virtual experiences.

From the first Logo program by Seymour Papert to Apple’s computers sweeping classrooms in the mid-1980s, technological evolution has changed the way we educate and assess students (Christensen, 2019). In the next decade, the Internet mushroomed connectivity globally with email communications, videos, images, text being shared freely. Prior to the lock down due to COVID19 pandemic and forced remote learning strategies implemented by universities and schools everywhere, educational technologies had already become popular. Digital citizenship skills, innovations and communication skills online were buzz words being used in professional teacher training to help prepare students for the fourth industrial revolution. Even United Nations’ 17 Sustainable Development Goals acknowledged the role of technology in a number of key areas, not just as a standalone goal #17 Industry, Innovation and Infrastructure, but as a key cog in helping to achieve all 17 goals, including goal #4 Quality Education (United Nations, 2020).

Artificial intelligence, Big data, Internet of Things or Augmented Reality, Virtual Reality and others - all cutting-edge technologies have been put to use in the education sector to enhance quality of education or make it more inclusive and accessible (Leiberman, 2018). Many theories have been formulated and put forward that encourage incorporating technology in teaching and assessing such as engagement theory, disturb learning theory, cognition, constructivism and many more that are meant to help “enhance and change students’ behaviour towards positive learning” (Clarkesite, 2016).

From learning management systems to proctoring and text-matching software, to activity tools and gamification platforms, technology has been supporting education for decades. Educational technology has been adopted within higher education because of its various affordances that align well with contemporary theories of learning such as social constructivism (Jonassen & Rohrer-Murphy, 1999). As stated by Roberts (2008) in the last decade, the incorporation (or the use) of technology in HE is mainly dependent on competition (amongst providers), student demand. Covid-19 has resulted in an environment that provides a third dimension which is the necessity. Unfortunately, technology is also frequently associated with academic dishonesty, particularly due to its pervasive characteristics and connectedness (Khan and Subramanian, 2012).

Academic misconduct is not a new problem (be it plagiarism, cheating in exams, getting someone else to do the work and so on). Bowers (1964) to McCabe (2001) and more recently McCabe et al. (2017) all self-reported cases ranging from 65% - 75% of students admitting to engaging in some form of misconduct. So it would seem that although technology makes it easier for students to copy and paste or contract cheat, it hasn’t had a considerable impact on the actual cases of misconduct over the years. In fact, Khan (2014) extensively showed the impact of using technology and how it helped more than hindered learning and upholding integrity among students. Technology in education is seen as an impetus that drives learning through the integration of multidimensional proficiencies, which encourage the development of transversal and transferable skills such as problem-solving, critical thinking and collaborative behavior (Gane et al., 2018). Therefore, it becomes necessary to utilize technology in our tutorials and lectures to foster active learning and apply the same when designing assessment tasks. The knowledge enabled through the use of technology can represent a variety of forms of knowledge and foster an appropriate level of competency that goes beyond rote learning and equips students with the skills required presently and in the future. According to Bearman et al. (2016), factors that require a careful consideration when designing assessment tasks are of contextual, individual and institutional nature, and the reconciliation and alignment of different thoughts that these factors enforce can be facilitated through the use of technology. Coupled with good assessment design, technology can help enhance student learning and reduce or minimize the likelihood of students cheating (Fang, 2012; Khan, 2014).

## Research objective

Although there are a variety of factors that influence a student’s propensity to cheat, this study explores how assessment design, redesign and inclusion of relevant technology can help to develop a culture of integrity and engagement. Particularly during the pandemic when schools and institutions tried to shift learning, teaching and assessing online, it is of utmost importance to learn from practical experiences to understand what practices yielded desired results. This research proposes to use experiential research methodology to document such experiences and develop an understanding.

## Methodology (reflection of our own and peer approaches)

This qualitative, exploratory study was based on informal mini interviews conducted through collegiate discussions and upon reflection of the authors’ own experiences as academics/external examiners (EE) who ensure the quality of assessment and grading in different higher education organisations in the UK, Australia and UAE.

We chose to use experiences and reflections as a method of inquiry for this study because reflections can help us to observe and be participants at the same time (Fook, 2011), enabling us to research and explore narratives of changes (Morley, 2014) made in the manner in which we structured our lessons and assessments in response to the pandemic. Reflection is most often thought of as a teaching and learning tool that helps professional learning (Schon, 1983; Coulson and Harvey, 2013; Nelson Laird et al., 2014). However, reflection has in fact been used as a research method to explore processes and experiences that can identify patterns in behaviour, thoughts, practices (Schon, 1983; Harvey et al., 2017). Bilous et al. (2018) have posited that reflection can be a valuable research method for developing curricula and for participatory research.

The discussions for this study involved academics from law, business, computing, biomedical science/medical sciences, and sociology. The discussions were based on:

- different assessments modalities (such as exams, short answer questions, course work etc) that are usually designed and employed in each institutions,
- the sudden impacts of employing “no detriment policies” in grading system to maintain fairness
- the effects of Covid-19 pandemic on the effectiveness of these assessment strategies,
- individual experiences of redesigning these assessments to mitigate the rigour of these assessments without affecting student experience.

**Fig. 1:**
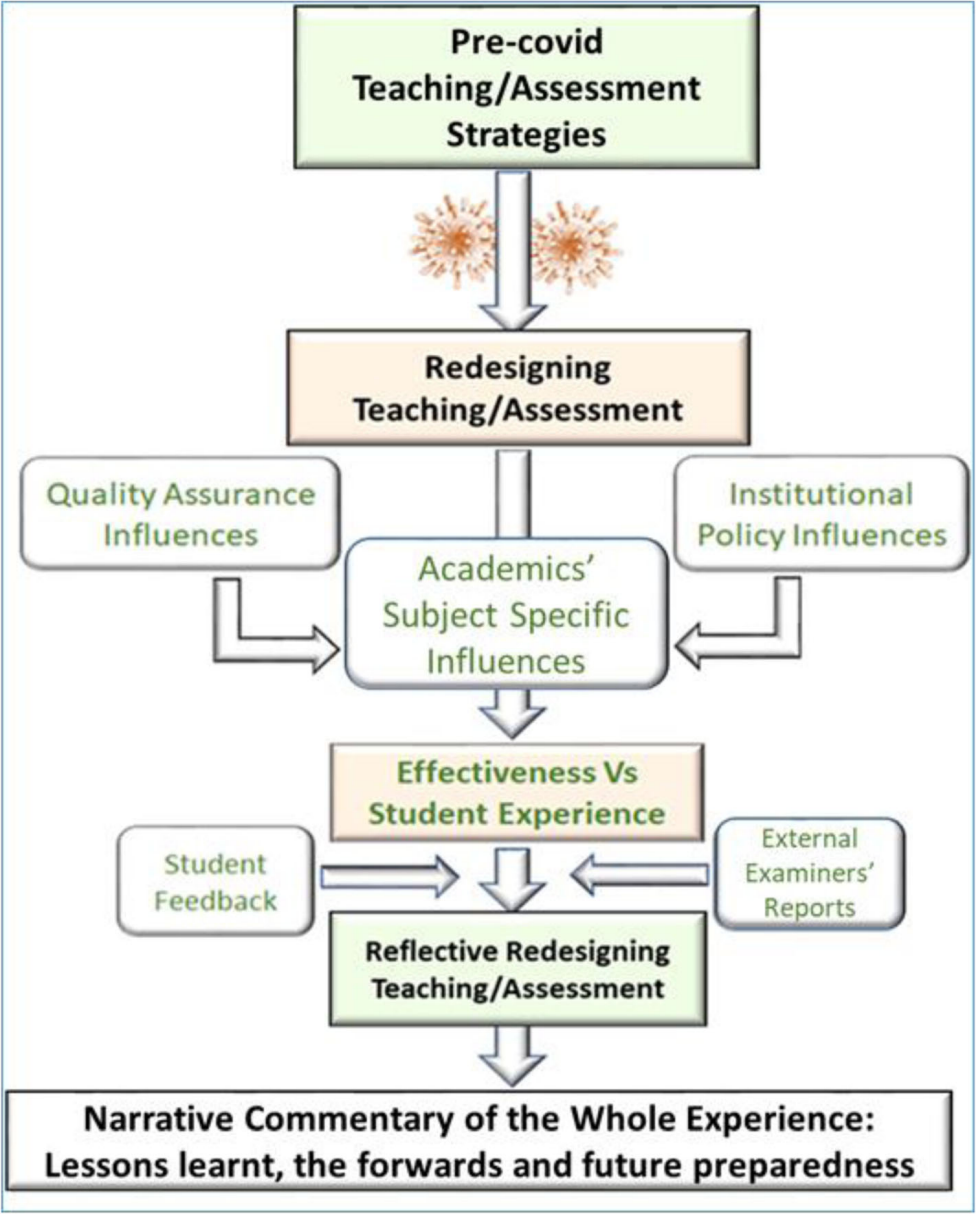
Schematic representation highlighting the reflective transformation of teaching and learning enquiry procedures during emergency distance learning

We also made use of a variety of modes for reflection such as visuals (screen shots), auditory (story telling), writing and activities (Bilous et al., 2018). Furthermore, we examined recently published and/or reported (in media) effective assessment strategies amidst pandemic. Finally, we tried to understand the effectiveness of these changes by writing (coding) our shared experiences which were summarised and narrated by the overall experiences of different academics and analysing them to identify patterns and good practices. Fig. 1 summarises the methodology/processes that are presented in this manuscript as a meta-cognitive reflection (similar to Eaton et al., 2019).

## Results and discussion

As explained above in the methodology, the study first compared the experiences of academics, external examiners, and quality assurance personnels to understand the immediate effects of Covid-19 related issues in higher educational organisations. We were particularly interested in the impact on (a) grading for 2019/20 cohorts, (b) adopting assessments from paper-based traditional examinations and course work to online grading, and most importantly (c) reflective changes that were made from feedback from students, external examiners, and quality assurance departments.

This is the narrative summary of our own experiences together with the findings from our conversations with our colleagues within the HE sector involved in this journey. Here, we will first show some examples of the chaos that were brought about by this pandemic.

## Sudden changes of grading system - fairness or madness?

In this study, we first looked at the “reflective transformation” of assessments and marking in response to the pandemic in March 2020. Our conversations with several academics in different HE organisations within the UK have highlighted that the universities were extremely keen to protect their students’ grading, especially the fact that they were forced to cancel some of their assessments. Many universities, hurriedly introduced the so called “no detriment policies”. However, authors found many inconsistencies which have cast doubts about fairness, equity and standard of grades and their implications. Some simply finalised the results based on the available gradings. Whilst others employed individualised approaches by comparing the grades from different elements. Yet others rounded up the grades to the nearest classifications. For example, those students who had an average mark between 67 to 74% were rounded up to 74% (i.e. First class). Also, as a quality assurance measure, these rounded up marks were clearly marked as “predicted grade” by inserting a ‘P’ sign next to it. Although it sounds reasonable, by showing this on the transcript, the potential employers would assume the original marks may have been as low as 67%. This disadvantaged the student who obtained 73%. This also created several student complaining discrepancies within grading resulting in unfair advantages as well disadvantages for some students. However, many universities listened to the students and addressed the EE concerns and readjusted their grading systems (based on no-detriment policies) as far as they could. In the authors’ point of view, this was the first impact on assessment integrity imposed by the pandemic.

## Assessing students amidst the pandemic - challenge or opportunity?

As for assessments or the ways of assessments are concerned, the academic community which are mainly used to traditional ways of paper-based methods were forced to use online tools and methodologies. This itself was a challenge as most of the academics were not properly trained to effectively use online tools for assessments and by the same time minimise the chances of any academic misconduct. Some transformed assessments into multiple choice questions, short answer style structured questions using online tools whilst others incorporated vivas, online presentations etc. These new methodologies, especially poorly adopted (or designed tests) caused severe challenges to upholding academic integrity.

For example, online MCQs and short answer tests would also provide the opportunities for the students to use common search engines to find the answer. Therefore, academics were forced to think about questions that would require knowledge application (rather than knowledge reproduction). Another problem was time management. Due to the pandemic and resultant lock-down, the universities were forced to deploy a 24 h window period for students to complete the tests. This of course provided the potential opportunities for the students to share questions (i.e. those who sat the text earlier provided hints for those taking the exam later in the day). This pushed the academics to randomize questions within a pool of question banks. During this process, the staff learnt to effectively use additional measures to minimise academic misconducts. In other words, many academics had a reflective learning experience about the challenges of these new methodologies. The institutions worked closely with higher educational quality agencies (such as Higher Education Academy) and those with university centers of learning and teaching that offered extra training in teaching and effective assessments techniques.

Covid-19 has resulted in reeducating academics to face the challenges of maintaining integrity by producing valid but variety for assessments strategies. Most importantly the community got together, shared good practices and tried to transform challenges into opportunities.

## Learning from mistakes and looking forward

One of the factors that influenced assessment design for many educators has been their concerns around academic integrity. Regardless of COVID-19, most educators in respective institutions have been reluctant to move away from traditional examination type assessments, even though studies have shown that examinations are least likely to measure students’ abilities to demonstrate real world knowledge, skills and attitudes nor does it ‘cheat-proof’ assessment (Dawson, 2016). Most institutional assessment policies and procedures tend to reinforce this belief by making it more difficult for innovative educators to try out different forms of assessments (Stobart, 2003). COVID-19 created an emergency where everything had to be moved online, even with all the concerns around academic integrity. Although a number of educators went down the path of using various forms of online proctoring and/or conducting vivas with all or a selected group of students, the courses that always had low levels of plagiarism instances had similar trends during COVID-19 assessments and vice versa. Although many educators transformed their assessments to non-invigilated types of assessment as a response to the COVID-19, the more successful initiatives were where the educators had already used some form of non-invigilated assessment prior to the COVID-19 related emergency.

By doing that these educators were a lot more comfortable in their approaches and were confident with the validity of their practices (Bretag, et al., 2019). One of the more significant approaches that has been discussed in various previous studies that can have an impact on reducing the motivation for students to cheat is the idea of personalising assessments to the individual students (Vehviläinen et al., 2018). Assessments that are engaging and personalised are less likely to be plagiarised, and often leads to more authentic demonstration of students’ capabilities. These assessments also instil in students that the educators trust their students to do the right thing and most students reciprocate this trust (Butler et al., 2016; Carless, 2009).

A great way to personalize assessments is through the use of digital storybooks. A digital storybook allows students to provide a reflective account of their learning through storytelling (Anand, 2020; McDrury, 2003; Price, 2012; Richardson et al., 2018). This encourages students to delve within their own experiences as a learner and curate a story that highlights their journey as a student and how they intend to apply their learnings in the future. Students need to provide evidence of any claims of their learning, using any freely available tools and resources available to them, online or offline. Often these stories are highly engaging and very personal and provide a realistic account of students’ learnings and application of these learnings, and are done within an authentic environment that can be shared with all relevant people.

Below we put forward examples of technological tools that were developed/adopted within authors’ own institutes to effectively assess students whilst maintaining academic integrity (see below).

## Technological tools to set up and assess in-class activities

In a traditional classroom setting, with umpteen amounts of physical resources including our own self we are at liberty to use them to maximize on connecting with students, engaging them with the content and pushing them towards deeper learning. A simple eye contact can prove effective in bringing a student’s attention back to the content. A spur-of-the-moment class activity, rotating students in the classroom and getting them to engage in a debate or poster making are all creative and student-centered teaching approaches that help teachers. However COVID19 pandemic forced us to move the entire experience online and while we may still have access to a lot of other resources, mainly the technology related aids, it is now more important than ever to understand our students’ learning styles, be flexible and understand how the resources we do have can be used in innovative ways. Most importantly, we should realise the fact that we have lost one important peripheral feedback - “the eye contact”; therefore, during the early days of remote teaching, the first course of action was to understand the students, their needs and challenges. Our observations yielded the following concerns students shared with the faculty informally:

- Feelings of isolation (this was perceived/reported by many academics, resulted in offering additional help)
- Newness of technology (some students were less acquainted with technology than we thought)
- Sudden increase in workload (repeated laboratory sessions due to social distancing influenced reduction in class sizes)
- Digital divide (managing students and staff with a variety of digital capabilities was a challenge)
- No time to understand own needs and barriers to learning (unpreparedness or the inability to change time managements according to lock-down situation)

Although students are technologically perspicacious, the whole experience of suddenly being online has been a challenge to them during the COVID19 pandemic remote learning. Emergency distance learning should not be confused or be used as a synonym of remote learning. Albeit congruently used interchangeably with remote learning, emergency distance learning is not the same as remote learning. During remote learning, faculty and students have umpteen amounts of time to design, train and deliver courses and assessments. In times of emergency, that is not always possible. Especially for students, the change has been drastic, to say the least.

**Fig. 2:**
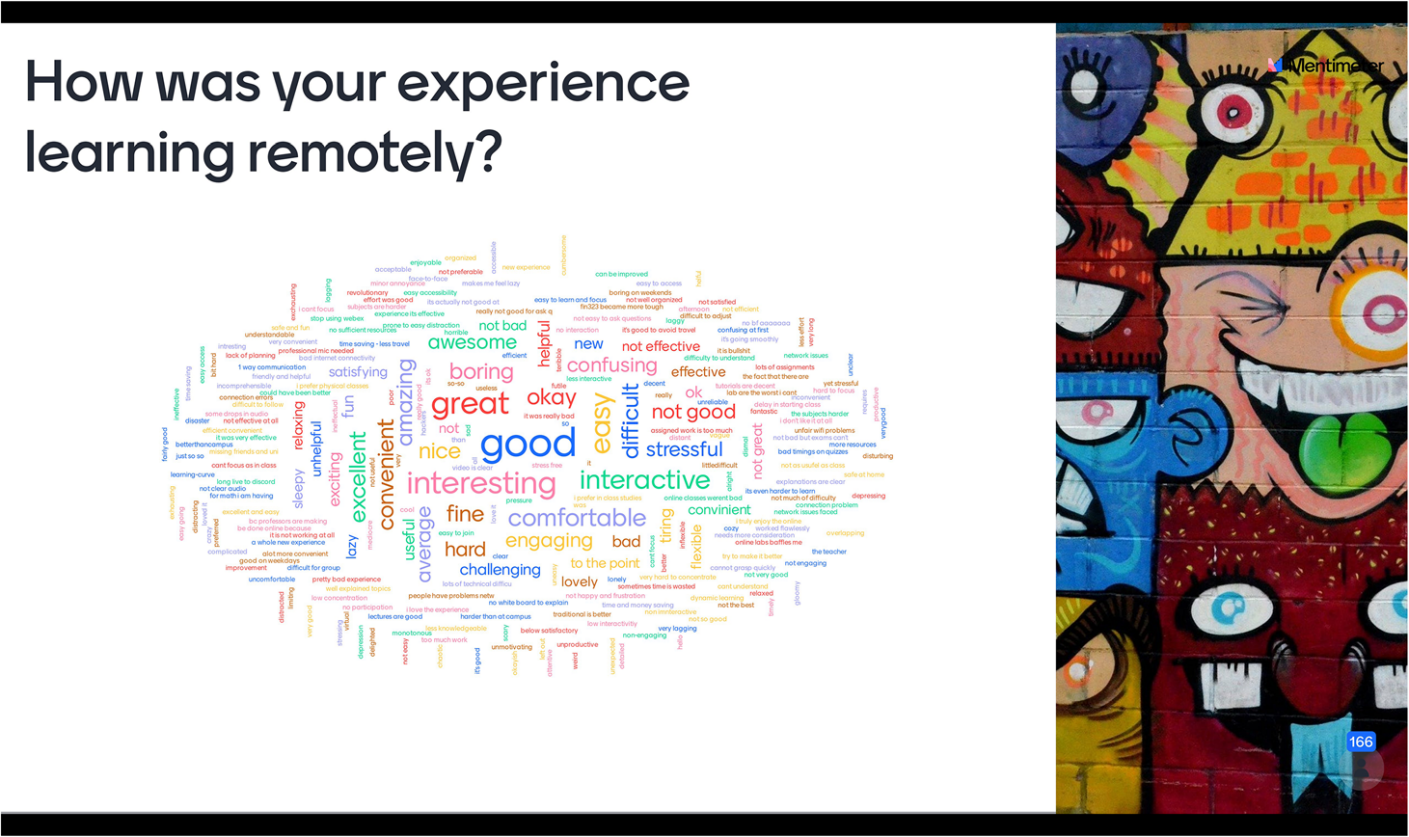
Word cloud illustrating how some students responded when asked to share their experience learning remotely

A word cloud of student feedbacks (below, Fig. 2) through social media on their experience learning remotely garnered the following word cloud which shows a lot of positive feedback, but also some negative such as “difficult”, “not good”, “harder to learn”, “technical difficulties”, “cannot grasp quickly” and so on.

As stated above in methodology section, we (the academics) researched and brainstormed on ways to use the technology we have at our disposal to see how we could best replicate the “traditional” classroom exchange and interaction of students and push them to higher order thinking even during remote space, thus minimizing likelihood of cheating or plagiarizing.

A number of technological services have been adopted such as Word Clouds, Padlets, Kahoot and even FlipGrids to address student barriers and help enhance their online learning experience. It is of vital importance that the technology chosen was simple, easy to use most often did not require any downloading or even account creation. Some of these are described below.

## The post-Covid journey - moving forwards with innovation and integrity

### Using word clouds to engage students with concepts

Word clouds are amazing tools that help to provide insight into student understanding, student thoughts and if used right, can be great conversation starters and help “visual learners process reading assignments” (Fuglei, 2020). Word maps are colourful arrangements of words in response to a thought-provoking question. There are many online services that offer to develop word maps for free. Mentimeter is one such service that we prefered to use.

**Fig. 3:**
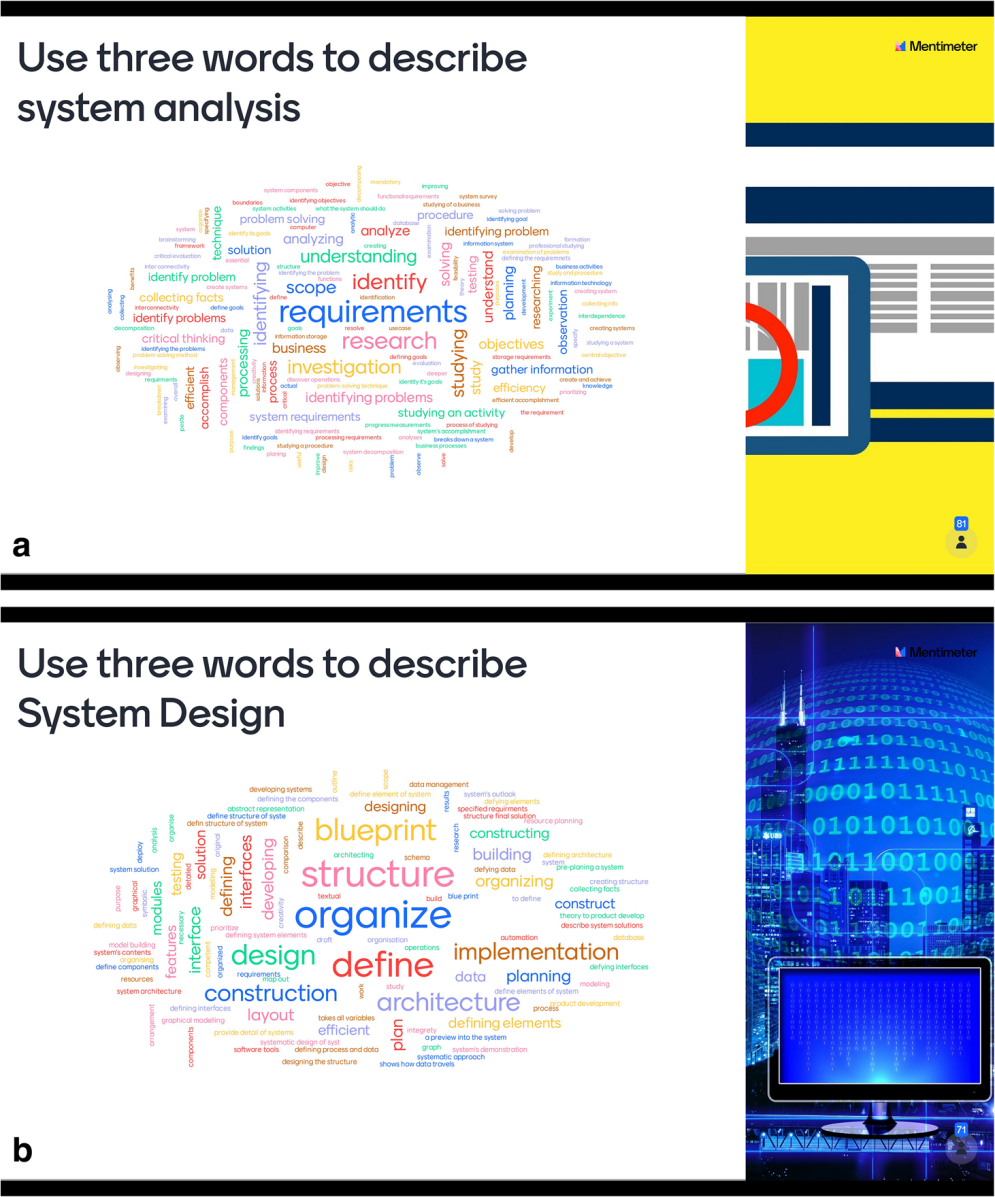
Example of two word maps created by students during System Analysis and Design course

Word maps aren’t just a collection of words entered, they make commonly used words or phrases in larger fonts, or darker colours too. We have found word maps to be exceptionally helpful in engaging students to visually see and reflect on a topic that we may have just debated or discussed in class. It is also quite exciting to use as most learning Management Systems such as Moodle allows for the Mentimeter to be added directly to the Moodle site for students to view the Word Clouds live. When teaching students about system analysis and design, for instance, we once used the Mentimeter Word Cloud (see Fig. 3) and got students to record words they could think about with each concept. This was in response to student confusion between the two concepts – that of analysis and design with it came to first year computer science students.

During the lecture, as part of the conversation, we asked students to use the Mentimeter and join in the exercise. As the word clouds formed, we shared the clouds on the screen during a live class and asked students to see what was being written which led to discussions about the difference between system analysis and design. This not only helped students visualize the concepts but also prompted memory and added to the level of cognitive engagement from previous lessons on the topics, leading to revision of the topics and allowing the educators to gauge overall understanding and preparedness of topic in class for students.

### Employing Padlets for class discussions and collaborations

Students feel isolated when they are engaging in remote learning. Remote classes can often seem like one-sided, archaic forms of “lectures” dumping information onto students.

Using technology is always a great way to introduce collaborative learning both in a traditional classroom and even online (Resta and Laferrière, 2007). In fact, we used Resta and Laferriere’s Instructional Motive Model (2007) to understand the complexity of using technology in collaborative learning which focus primarily on four areas

- collaboration skills and knowledge creation
- engagement in knowledge creation
- cognitive performance
- flexibility of time and space

We found Padlet, an online, free service that acts as a cork-board in the virtual space to help in contributing to all four instructional motives. Padlet is a multimedia service that allows live interaction between student-peers and between students and teachers.

It allows not only text, but videos, images, flow-charts etc. to be posted on what looks like a cork board and invites collaboration easily and effectively online. Likewise, university managed learning management systems such as Moodle allows the code to be embedded directly into it so students are continuously engaged on familiar ground when using these tools.

**Fig. 4:**
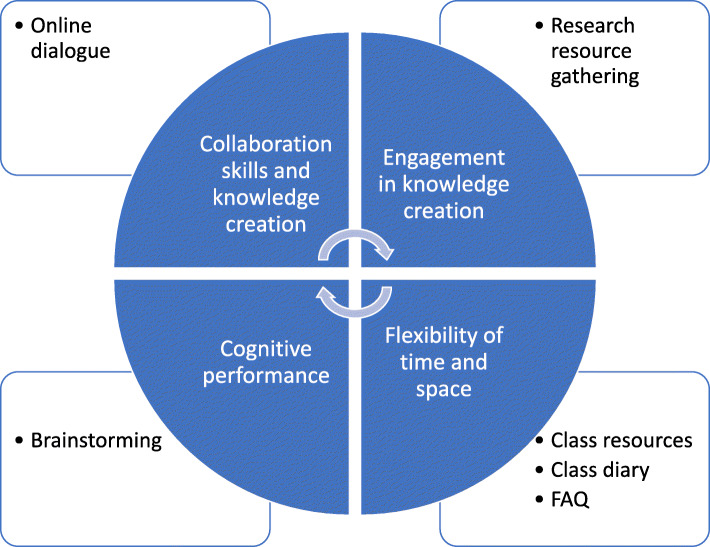
Use of padlet to contribute to the four instructional motive (Source: Beltran-Martin, 2019)

**Fig. 5:**
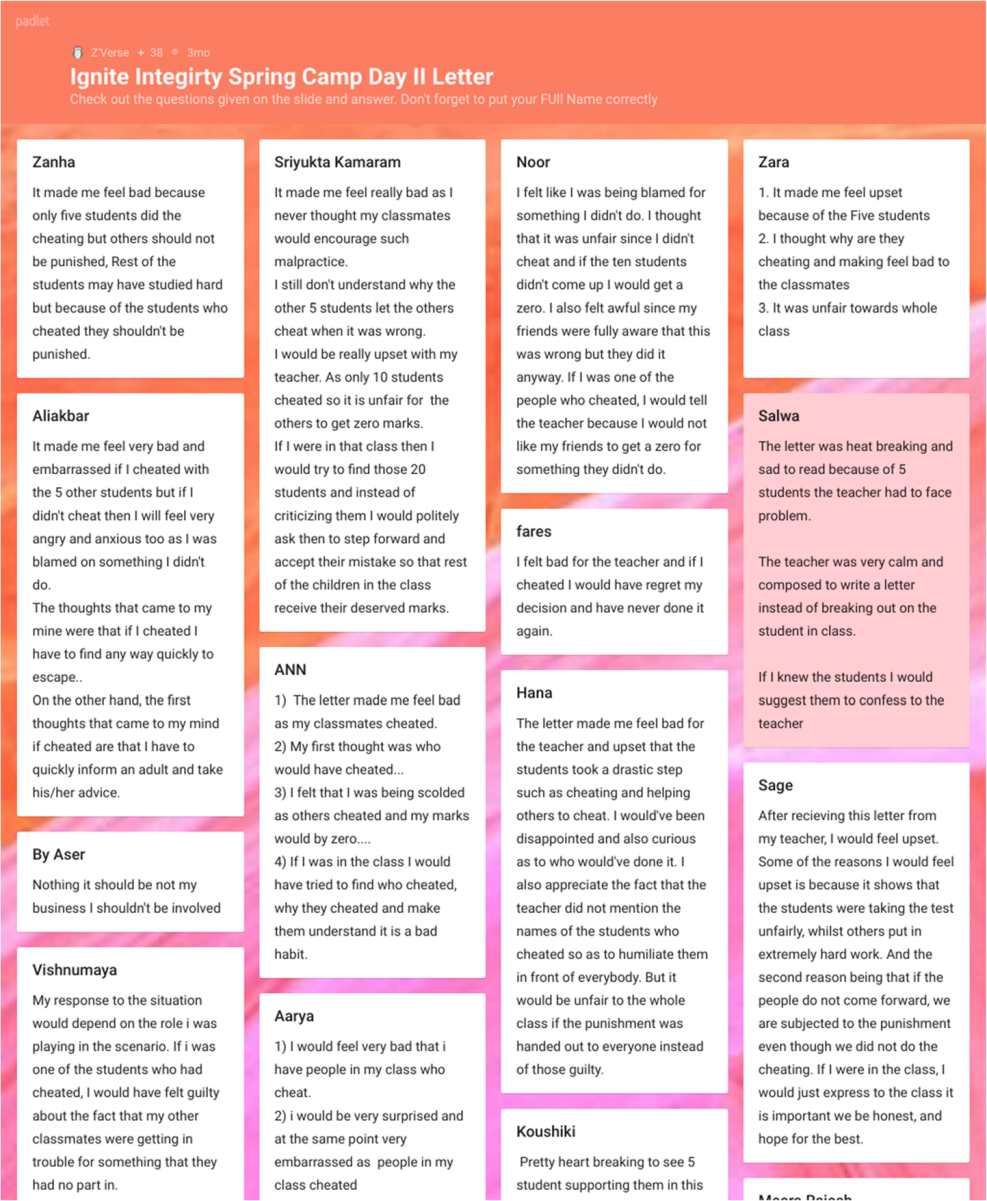
An example screenshot effectively using Padlet canvas to create collaborative discussion connections

Using the Beltran-Martin model (2019) (based on the Resta and Laferriere (2007) - see below), we were able to successfully use Padlets to design collaborative activities around subjects such as discussions, gathering answers to questions, brainstorming sessions for research, asking what students found to be easiest topic and hardest for each lecture, creating live question banks, even allowing for students to create their own portfolios online for their projects (adding research, links, images, articles for projects) and so on. These are summarized in Fig. 4 below. For example, for a first-year information system subject (that taught students the basics of information systems, steps to developing software by analysing existing systems and designing proposed solutions), we asked students to research and answer questions pertaining to concepts taught in the class and applied to a real-world example (see Fig. 5).

As a graded activity, students were able to work on tasks, discuss with peers and write down their findings, then further discuss the answers in the live tutorials. This allowed for students to join in knowledge creation, cognitive performance and gave them flexibility of time and space. Padlet also allowed the educators to download the student feedback either as a PDF or an excel sheet to be graded at a later stage.

Padlet allows for different formats to be used to set up an activity, from a cork board post to canvas (with connected posts), to location on maps, to timelines, even conversations. With the ease of adding images, links and videos, a Padlet activity can allow students to choose a format of engagement that aligns with their learning styles. Using this technology saw a marked reduction in stress and anxiety over assessments in the subject, and increased student overall engagement.

### Adopting Flipgrid to increase accessibility and inclusiveness

A major issue with remote teaching has been the ability to provide equal opportunity for all students to participate, engage and interact. While the traditional digital divide concept has always been about those who have access to technology and those who lack it, especially during the emergency distance learning, the term has taken a new meaning. We had students who were not in-country anymore, stuck outside their institutions, in their own countries during the lockdown; or students who came from conservative moral backgrounds and could not use their webcams or even mics during live classes; or students who lived in shared spaces and could not use their webcams or mics to join in class discussions, answer questions and so on.

Upon some research, we came across Flipgrid, an online free-to-use video sharing tool that is classroom friendly. This technology allows students to make short videos easily, sometimes even without the need to create an account! “Flipgrid is a response system that allows students to explain or show their learning using video” (Clark, 2020). The technology allows students to provide responses, present their ideas even if they have missed a live class or been unable to connect or not allowed to use a webcam during class. We successfully used Flipgrid for our ethics course as well as more technical courses. Students have used the service to prepare short videos explaining their ideas and solutions to entrepreneurial pitches, ethical role plays replicating a courtroom debate on intellectual property, system design problems, issues of system analysis and more.

**Fig. 6:**
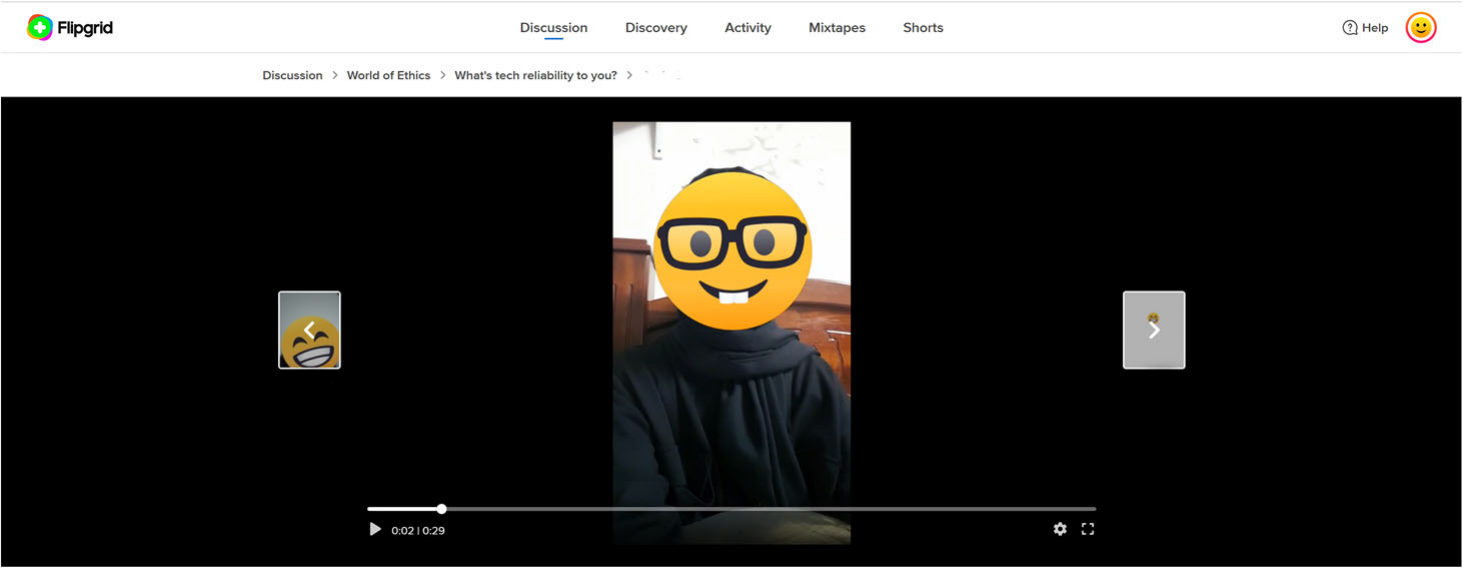
An example screen-shot of Flipgrid allowing students to choose “privacy” when making videos

Flipgrid has levels of privacy that allows a teacher to set it up as per class requirements. The video formatting is easy with a host of features such as editing, trimming, adding audio, taking selfies and more, and allows students to “hide” their faces by blurring them or placing “smiley” on them if students are conscious (see Fig. 6). Flipgrid also allowed authors to set up student “comment” so they could reply or “like” each other’s work.

### Kahoot! – gamifying assessments and activities

Despite efforts, it is sometimes still difficult to reach all students attending a class, particularly when teaching a technical course. One way to bring “class” feeling into a virtual class is to use gamification. Gamification is when game elements are used in non-game contexts that increases participation, excitement, engagement, and healthy competition (Lee & Hammer, 2011; Smith, 2014; Packt, 2019).

Kahoot! is a gaming tool for classrooms that we have been using regularly in our subjects. A free service online, it allows teachers to set up fun quizzes using various formats such as MCQs, T/F and so on. The platform presents the quizzes as games, awarding points to students’ answers based on correctness and speed at which they answer. We are able to control the question text, any images and even the length of time and points for each question.

Designing a Kahoot! is easy but not always straightforward. It is important to realise the idea of such a game isn’t necessarily to test really difficult concepts, although it can definitely be used for that too.

**Fig. 7:**
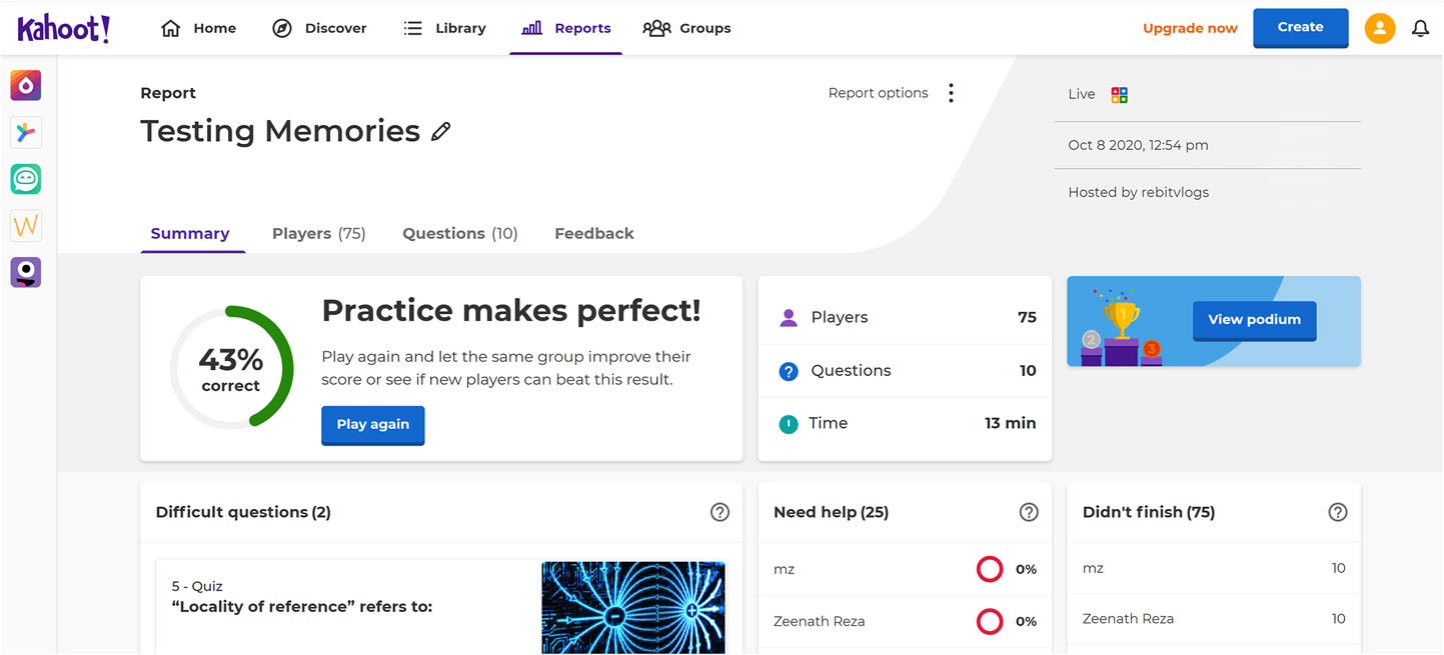
An example dashboard showing analytics produced by Kahoot for a game played

Kahoot! allows teachers to set it up as a live game competition that can be shared with a live class or as a challenge for each student to try at their own pace. When administering a Kahoot! we do not just let the game be played by students; we dawn the role of a game-show-host with commentaries, jokes, laughs, and sometimes stunts to make the game a “live” experience. Calling students who are getting answers right, encouraging students who are falling behind are some of the ways we have found we can keep all students’ interests in the game. We used Kahoot! not just as a quiz that was graded, but also as a recap tool at the end of a lesson and as a personalized learning tool for students to go back and access and try out again and again as revision. In addition the application provided analytics such as which questions were least answered, which questions were most answered, which students had difficulty answering, etc. (see Fig. 7). This helped students develop confidence in the knowledge they learned and made it less likely that they would look elsewhere for answers.

### Instructional videos

To ensure students were aware of how to use the technologies we introduced as part of learning or assessment with instructional videos of all technologies or teaching techniques and assessments that we designed, used or implemented in our classrooms. Simple videos made and uploaded to a private YouTube channel and then made available through Learning Management Systems (LMS) seemed to decrease students’ anxiety over using the various teaching tools and participating in the assessments that we introduced.

Videos on netiquettes, how to engage with lectures online, how asynchronous assessments work, how to upload assignments using LMS database, Mentimeter, Padlet, Flipgrid were some of the instructional videos created to support the techniques on the online platforms.

### Industry-based assessments

One of the proactive approaches to instilling integrity values into students and fostering a culture of integrity beyond a course is producing creative assessments that help push students to higher thinking. These assessments give them confidence in their knowledge application and provide them with satisfaction of knowing they can apply the knowledge in real world settings. This set of educator-developed assessments allowed students to be the knowledge content creators and assisted the academics to select formats of assessment that tested their knowledge in the best possible way. Upon researching and studying teaching methods and theories, these educators designed and conceptualized two programs using Collaborative Learning theory and the Experiential Learning Theory.

Collaborative learning theory involves student-student (peer) interactions that allow for greater learning/deeper thinking, better communication, leadership/ team management and spirit (Vygotsky, 1962), while experiential learning theory emphasizes the role of “experience” in the learning process (Kolb, 1984). In fact, experiential learning theory is sometimes considered as Problem-Based learning which calls for students to be central to their own learning process.

The aim with the two programs was to invoke curiosity in the students, provide guided discovery opportunities through mentorship and hopefully result in their critical thinking about problems and solving them (Bates, 2019). The details of the programs and how they were modified to adapt to remote delivery are explained below.

#### ReBitVlogs

One author and their subject coordinator conceptualized and implemented a student program called ReBitVlogs – Responsible Business and IT Use Video Blogs program (ReBitVlogs, 2020). The program aimed to initiate, enhance and engage students on responsible business and ethics of the information age across campuses. As an innovative platform that was initiated to engage students in discussions on pressing responsible business and technology-use issues, ReBitVlogs is a link between students and academics with industry experts, government and non-government officials, creating an environment of awareness, facilitating dialogue towards a sustainable, responsible future.

Originally initiated in 2017, the program grew to now be in its 10th iteration. It was really useful during the pandemic for students to come “face-to-face” with industrial experts online. Upon selecting a theme, the first part of the program is to run a story-telling competition through posters. Winning teams are then invited to join industry experts in a panel discussion to continue the conversation. During the emergency distance learning, the program continued with online storytelling and poster competitions. Students were encouraged to find creative ways to present their “stories”. Electronically designed posters using simple Word or Powerpoint to using Adobe Illustrator and even online tools such as PiktoCharts and Canva.com, students got creative and produced fascinating stories around chosen topics.

**Fig. 8:**
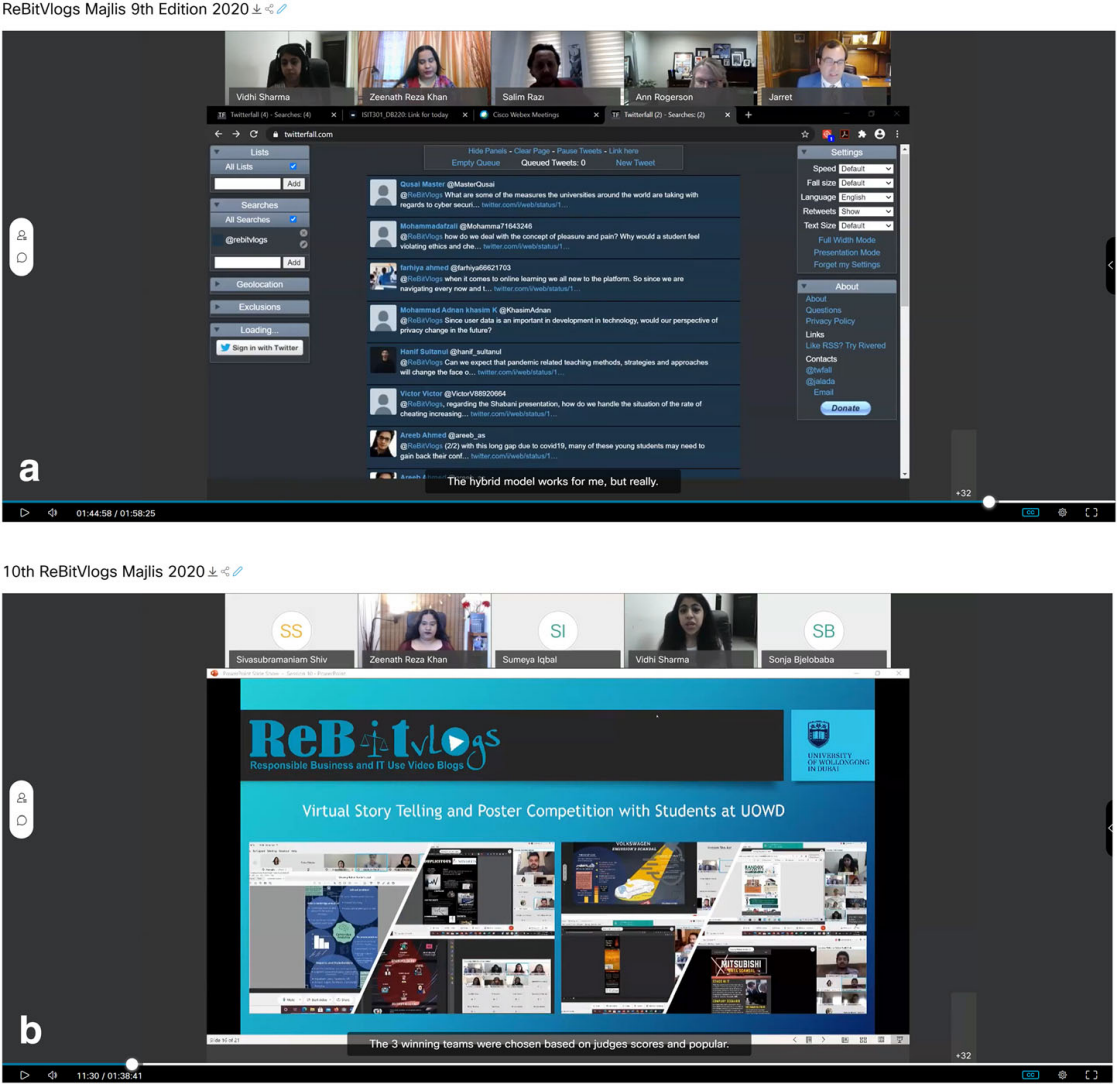
An example ReBitVlogs online Competition, using Webex and Twitter Fall to engage students

Going virtual helped make the online panel discussion (*Majlis*) international with experts from Australia, Turkey, USA and Europe taking part in the discussions and engaging with the students. Invited industry experts found the students to be highly engaged and well researched. Students were excited to participate either as panelists or as audience. Audience got to use Twitter to ask questions live to the panelists which were showcased on Twitter Fall (see Fig. 8). The projects reported zero cases of plagiarism.

#### WISP: real world innovative solutions to real world problems

In teaching the importance and methods of system analysis and design for Computer Science students, educators conceptualized WISP – real world innovative solutions to real world problems (WISP, 2020), a program that invited the university’s alumni to join a “Human Library” (Human Library Organization, 2000) as mentors to help first year students understand how the real world works, what kind of solutions are expected and how important these skills are that are taught so early in their degree. This “library” of human experts and mentors were accessible to the teams taking the subject for the duration of the program. Teams were then able to “book” experts from the library on “loan”, much like a book from a physical library, for a small period of time based on level of expertise and time the human experts were able to dedicate. The human library has been built upon partnership with industry experts and companies through the university’s Alumni Relations Office and Marketing Department.

The program deliverables included:

- Student-initiated and proposed solutions that are approved and appreciated by industry experts
- Possible on-the-job training opportunities for excelling students
- Possible short-term internships with partnering industry experts and/or their network for excelling students
- Corporate Social Responsibility opportunities for industry partners

Student teams were matched with Human Library experts for mentoring programs, and ultimately teams presented their “pitch” to the judges/experts, convincing them of the proposed solution as “the” solution for the problem identified.

**Fig. 9:**
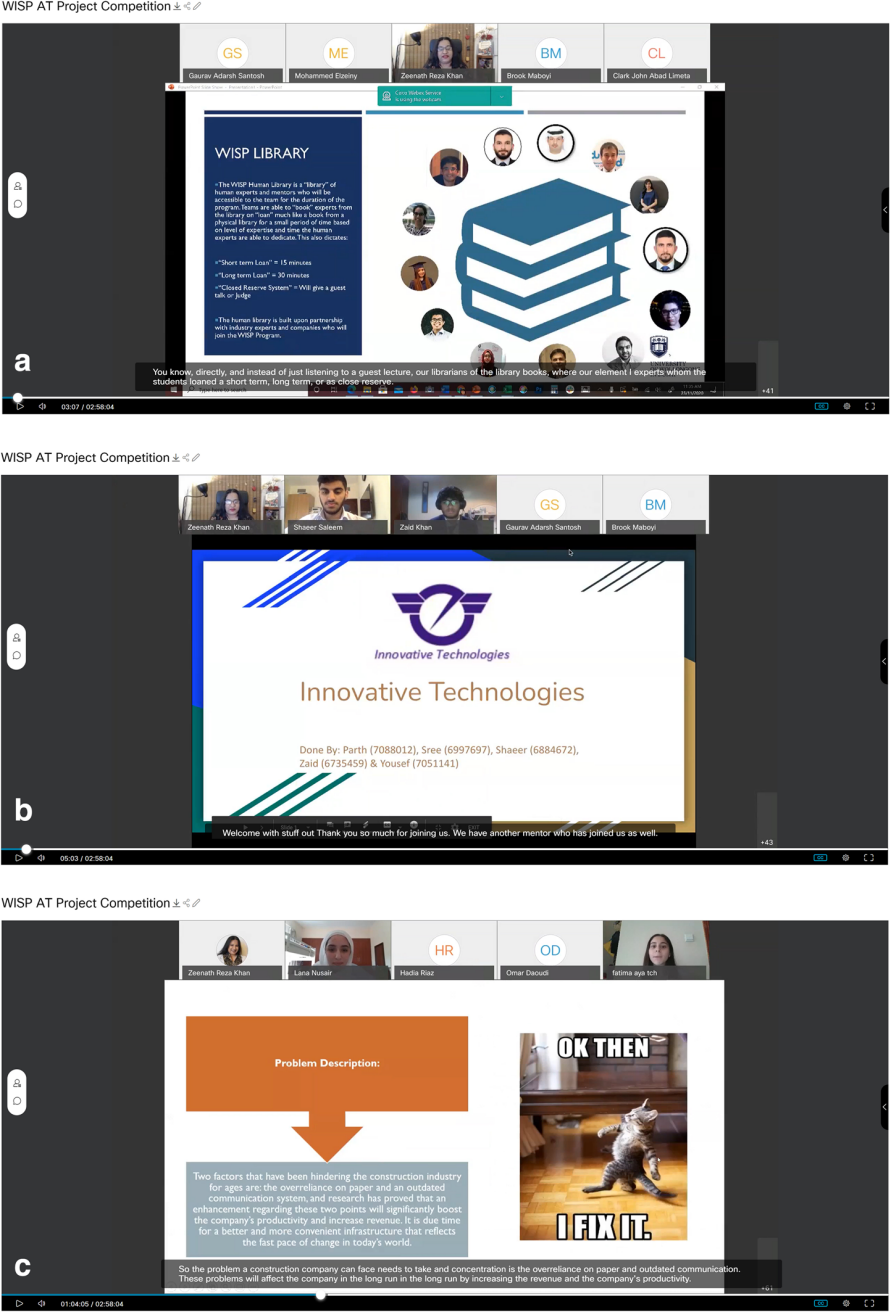
An example screen-shot of WISP Human Library and its usage with mentors

This is yet another program that utilized the virtual platform to connect students to their peers and to mentors, reducing their anxiety and stress, to focus their attention on engagement with subject content, knowledge creation, collaboration and enhance their overall learning experience that is expected to help them throughout their degree program, well beyond the subject (see Fig. 9). The projects reported zero cases of plagiarism.

### Using online debates to enhance peer interactions and engagements

Some of the authors within the medical field were forced to transform their usual debate-based learning activities as online activity. Although it was perceived by the academic as “*mission impossible*”, using online learning tools such as Microsoft-Teams, Zoom, and WebEx, these debate-based activities have been successfully delivered. The number of participants for these activities differed amongst institutions, ranging from 35 to 255 participants. For larger participant numbers, WebEx was found to be better suited. Also, many platforms allowed devising small discussions by splitting the participants into different groups in a timely fashion and then rejoining them to report back in a plenary session. Most important observation of using a web platform for debate-based learning was the enhanced student contributions. Those students who were usually silent during face-to-face delivery, were commenting and taking part in the discussions.

Others simply wrote their opinion and observation onto the discussion board which were picked up by the conducting academics and addressed. In fact, this is another unexpected positive outcome from emergency online learning.

## Summative analysis of shared experiences

Maintaining high levels of academic integrity in academic environments has always posed challenges for educators at all levels of the education ecosystem, but particularly within higher education. This is because these learners are often a lot more independent and focus around their study processes and engagement. Educators have explored various forms of assessments to assure learning outcomes, and although there are numerous examples of innovative and engaging assessment practices, most educators tend to rely heavily on invigilated examinations as a tried and tested means of assuring that the work that students submit is their own (Dawson, 2016).

Increasing use of technology within higher education has provided opportunities for educators and students to engage in diverse and unique learning activities and topics. For example educators are able to use relevant technologies to engage students through immersive simulations, facilitate collaborative activities with students from different continents to solve complex real world problems, and so on. Technology is also increasingly used in various forms of assessments. It has unfortunately also provided opportunities for some students to engage in various forms of unethical behaviour (Fawns, 2019). This is particularly so within education where some students try to take advantage of the ever-connected nature of online activities and at times the perceptions of anonymity to cheat and therefore get credit through someone else’s effort. The omnipresent technologies such as smartwatches, smart phones and other emerging technologies enables many students the ability to engage in various forms of unethical behaviour.

The COVID-19 pandemic forced all educational institutions to become a lot more inventive in most of their teaching, learning and assessments procedures. Although teaching and learning during this pandemic has been challenging for educators and students, the most significant challenge for educators has been designing assessments to maintain academic integrity. Most assessments had to be carried out online, and in many cases they were non-invigilated assessments. Even institutions that used various forms of online proctoring technologies and services were challenged to assure academic integrity (Bretag et al., 2019; Daniel & Thomas, 2019; Dawson, 2021). For example, Dawson outlines a number of ways in which motivated students could ‘hack’ online and face-to-face examinations regardless of the presence of any invigilators (2021).

Maintaining academic integrity should be a multi-faceted approach. Although it is widely accepted that plagiarism and other forms of cheating cannot be completely eradicated, there is enough evidence to increase confidence that with appropriate assessment designs, redesign of existing assessments, engaging classroom and assessment practices and constant awareness about the importance of academic integrity it is possible to discourage it.

It is widely recognised that written, invigilated examinations may not be the best way to assess many types of knowledge, skills and attitudes that students need to have in order for successful work after studies, however many educators in higher education are reluctant to move aware from examinations (Hillier & Fluck, 2013). Although COVID-19 created the emergency for everyone to relook at their assessments and deployment strategies, most responses during this pandemic was reactionary rather than proactively planned. However, COVID-19 has provided an opportunity to explore other ways in which we can continue to uphold academic integrity in varied assessment and teaching modalities.

Although many studies have evaluated and suggested a number of different types of academic integrity verification tools, where students’ work is cross-checked against other work on the Internet (Ellery, 2008), in this paper the authors have attempted to explore other ways of upholding academic integrity. Approaches that attempt to identify plagiarism and cheating may be too late in the student’s assessment cycle and therefore may not effectively play an educative role on developing ethical behaviours that encourage learning rather than focusing on the final grade.

The authors have leveraged studies that demonstrate the significant influence of various engagement strategies to improve student outcomes to develop effective engagement strategies for online student cohorts (Nelson et al., 2014). Although the online mode was imposed upon all higher education institutions around the world, it also provided us with opportunities to explore appropriate technology tools to engage students. Technology became even more important during COVID-19 as an enabler to engage students in meaningful activities and develop engagement. The various examples of assessment design and student engagement focus on using easily accessible technology tools that are in most cases free to use or at worst free for the students to use. Technology tools should also seamlessly integrate into existing teaching and learning approaches, such as supporting classroom discussions, and accessible from standard equipment like smart phones.

Similarly, assessment designs that actively discourage unethical behaviours and scaffold students into effective learning should also be encouraged as many would then help students acquire more transferable skills. The COVID-19 emergency that all educational institutions had to address has spawned many good practices initiatives that we can learn from going forward, pandemic or not. Many of these strategies have been discussed in the sections above.

## Addressing exceptional extenuating circumstances

Emergency distance learning did pose some unexpected (or unpredicted) challenges too. The first issue was “digital literacy” of the students. Most of these online based approaches were new, or newly adopted using tools that were neither introduced to nor practiced by the students. Explaining “how to” use these tools via an internet-based environment was a challenge. In contrast to the general academic perceptions of all HE students being experts in using online tools, many students were not competent enough to use these tools. This may be due to a variety of reasons (a) unfamiliarity of educational online tools (as students usually use more games/game-related IT tools, (b) mature students (who hardly use online), (c) sheer inadequacy of students to understand algorithm of these packages (especially to those complicated on-line packages that replaced laboratory-based learning) and (d) accessibility of these packages (i.e. the inability to run these highly sophisticated packages in their home countries). This was also recently highlighted in the Times Higher Education article stressing about carefully adopting “educational technology” to meet learning goals (Losh, 2021). Losh further highlighted that academics need to be mindful of the variety of component prerequisites of digital literacy including (a) technological aptitude, (b) social confidence, (c) privacy awareness and (d) financial ability (2021). However, in actual fact the sudden nature of this pandemic did not provide time to adopt these technologies focusing on students aptitude on above said components of digital literacy. In our experience, this has created more work for academics, first to introduce and make the student understand how each of the online tools works (through instructional videos, as explained in prior sections), before actually engaging them into the subject specific learning. Some of us received assistance from the respective IT departments in the form of skills development workshops for students and academics.

Second issue was the dependency of the internet with a reasonable bandwidth to carry out and receive these online sessions. These are mainly experienced by the students as “receivers”, especially during the lockdown periods when the majority of students have moved back to their hometowns or countries of origins (in case of international students). Some students did not have their computer facilities at home, others (especially the international students) faced financial restraints to obtain internet access, or happened to share the wi-fi with too many users in the same space. The former was somewhat addressed by the universities by lending institutional laptops on loans. The latters have remained as sources of problem (in fact a limitation) against ‘e-teaching/assessment’ strategies. In 2020, the UK government had taken some initiatives to provide free internet access in collaboration with major mobile companies to support students from financially impoverished school students (Difn, n.d.). Similarly, the UAE Ministry of Education and Telecommunication Regulatory Authority worked with the leading internet providers of the country to give free mobile internet access to families who did not have home connections to continue emergency distance learning (Abbas, 2020). However it is not clear whether this was also provided to the students in HE; or in other countries.

The third concern was students losing their “sense of belonging” in the context of a university community, which has always been an important factor in creating inclusive learning environments. We as authors, despite our varied fields of expertise, experienced the student losing their “cohort identity”, especially during lockdown when all activities were online. As described by Mooney and Becker (2021) “belongingness” not only influenced students’ backgrounds but also their interactions and experiences with other students, academics, technicians etc. The lockdown has resulted in them losing the physical environments. Whilst some students maximised their virtual interactions with their peers, others struggled to cope with these sudden changes. This may be one of the contributing factors for increasing mental health related illnesses amongst HE students during Covid-19 (Marelli et al., 2021; Meda et al., 2021; Savage et al., 2020). Considering the lockdown was slightly relaxed, some UK universities have offered limited on-campus activities in STEM subject areas. One of the authors’ reports, his institution has tried to introduce a blended applied learning model where students would receive a minimum “3-hours per student per week” on-campus activities. However, it was observed that there existed mixed engagements from the students, around 85% of the students actively engaged with this model, the rest were not prepared to engage in on-campus activities (unpublished data: calculations were made from the number of remote study requests received). Although this data is from one institution, representing one discipline, this may be due to (a) the worry about Covid-19 infections, (b) international students unable to return (as stated above) and (c) medical/shielding related issues. Also, the ever-changing Covid-19 related restrictions and regulations hindered almost all universities to provide a consistent approach. Overall, our approaches were constantly challenged by the changing situation. Yet it did open up new and innovative ideas for effective learning and teaching activities.

Finally, and perhaps the most stressful issue that we were forced to rethink, or address was related to the potential academic misconducts in online assessments. Almost all HE institutions were concerned about this. During the pandemic we transformed our traditional assessments strategies to a variety of different online based evaluations. These included time-released or time constrained assessments (TCA), take-home examinations (online essay type questions without any time limitations), video-based assessments (including on-line viva voce’s), online student presentations (oral and poster), on-line structured essay questions, short answers, multiple choice questions (MCQs)/multiple response questions (MRQs), extended matching questions (EMQs) etc. (Gamage et al., 2020). Our own institutions have also employed these measures depending on subject areas, and the levels of study. Each of these have their own advantages and disadvantages. In the context of this manuscript, we concentrated on selecting the most appropriate mode of assessment(s) to minimise the chances of cheating. Here we narrate some initial “threats” against integrity and discuss some possible way forwards.

Eaton (2020) summarized the pandemic and its resultant online delivery as having provided opportunities for commercial file-sharing and contract cheating companies to thrive. This is particularly true in take-home assessments where the students would have a window period with a deadline to submit their essays/work. In our experience, there are two ways to handle this (a) using structured essay questions or EMQ’s (instead of providing essay type questions) and (b) employing feedback led continuous assessment strategy (a measure pioneered by one of the authors). The former is useful in applied and/or STEM subject areas where knowledge applications with different context is expected. The latter can be employed by any subject area as it requires a continuous online dialogue between the students and the academic. However, it is time consuming and can only be employed in a smaller cohort of students. With respect to online examinations, the main threat is the students’ accessibility to the internet (as they are using it to answer it). Students often “Google” the question and answer. In this context, some academics and institutions have used time restricted structured questions (where each question would impose a time duration to minimise searching answers). However, this would also minimise the chances of making sure the student understands the subject area (as the answers would often be superficial). Another method of minimising academic misconduct in online examination is providing questions that need knowledge application in a different context (rather than reproduction). This is particularly successful in STEM areas of HE but may not be suitable for concept-based education. On the whole, we, like several academics, have experienced different assessment related challenges that are linked to maintaining integrity. In our opinion, we all need to move away from the notion of “assessment for acknowledgement (or reproduction) towards assessment for application”. Academics also need to refrain from recycling the past question or using publisher databases because question recycling would enhance file sharing practices; instead, we need to be innovative.

At this point we would like to point out that the experiences about exceptional circumstances and our ideas of maximising integrity during online examination, are purely narrative. The pandemic started only in 2020, so we are only sharing our experiences from online teaching. As for the challenges of adopting and applying these innovative technologies enhanced learning activities during this pandemic, we can safely say these approaches (stated herein) may not be a perfect solution but they have paved the way to continue to deliver our courses achieving the programme learning outcomes.

## Concluding remarks

Covid-19 pandemic did impact the ways of learning and teaching in higher education. As a sector, we faced similar challenges across institutions and countries. Whilst some of the academics struggled to acclimatize to this new online based teaching/assessment environment, many of us transformed this challenge into opportunities for innovative teaching and assessments.

Moreover, with the help of institutional learning and teaching excellence centres and through collegiate sharing of good practice, we believe, we can help each other to enhance integrity with effective practice in our online and face-to-face classrooms.

Faculties from different institutions shared their collective experiences during the pandemic that highlighted similar challenges and solutions that were developed in response to the crisis. This provided an opportunity to learn from one another, with particular focus on on-going good practice to uphold academic integrity beyond the emergency distance learning experiences. As Donald Rumsfeld (the former US secretary of Defense from 2001 to 2006) once stated “*there were known knowns and known unknowns; also unknown unknowns*” (Rumsfeld, 2002). As academics we learn from these variables, reflect and maximally adjust to enhance the learning and teaching activities.

Authors are confident this paper will add significant value to the body of existing literature; however, it is important to note that the study is limited to the experiences collegially shared and reflect measures taken under force majeure and therefore the findings made should not be generalized across the sector.

## Data Availability

All data is available upon request.
